# Supplementary material for: Major depressive disorder and bistability in an HPA-CNS toggle switch
Source: PLoS Comput Biol. 2023 Dec 6;19(12):e1011645. doi: 10.1371/journal.pcbi.1011645 (PMC10699645; doi:10.1371/journal.pcbi.1011645)
Supplement: Attachment — Submitted filename: MDD and bistability paper - revision response.docx [file pcbi.1011645.s013.docx]

Dear Editors,

Thank you very much for the referee reports and for your positive consideration. We have addressed all of the referee comments. We added a new table, added sensitivity analyses, clarified terms and added references to biological support of the assumptions. We improved the figures and fixed typos. We believe that our revised manuscript is improved in terms of rigor and clarity.

Below please find a point-by-point response to the comments.

Sincerely,

Uri Alon and Ben Ron Mizrachi

**Point-by-point response:**

Reviewer #1: "Major depressive disorder and bistability in an HPA-CNS toggle switch" (PCOMPBIOL-D-23-00795) by prof Uri Alon and colleagues submitted to PLOS Computational Biology is a well written and important paper continuing earlier contributions by the same authors.

Response: We thank the reviewer for this endorsement.

Overall, the core part does only require minor changes whereas the Method section and especially the presentation of the mathematical model and the analysis of this needs extensive work.

P.3 Results has a subsection named ‘A minimal model of the HPA-CNS toggle switch’ but in the Method section this is denoted the MDD model. You need to decide upon a single name! Despite your choice an appropriate and more descriptive heading could be ‘A toggle switch model of the HPA-CNS system’ (In the text the ‘minimal model’ feature is mentioned).

Response: Thank you for the comment. We now choose the name HPA-CNS toggle switch model, or briefly the HPA-CNS model. We accordingly revised the Methods section. We revised the heading as suggested to “A toggle switch model of the HPA-CNS system”

We changed the sentence in the method section from “to obtain the MDD model” to “to obtain the HPA-CNS model slow time steady state solution”, to describe more precisely equations (15-17) in the method section.

P.3, l.-5 (meaning line 5 from bottom of page 3). I suggest substituting the word ‘described’ by ‘understood’.

Response: We replaced “described” by “understood”.

P.4 and throughout the paper, the imprecise and mathematically misleading term ‘steady state’ is used for ‘quasi-steady state’ (which could be abbreviated QSS). Please correct this throughout the text.

Response: The paper has two types of solutions- the fast timescale quasi-steady states, and the slow time scale steady-states which are a steady state solution of the full set of equations with the fast and slow variables. We went over the paper, and wrote the appropriate term in each case.

P.4 , l.-6 ‘Kd’ is not defined. You may explain it in words.

Response: We now define Kd as “the dissociation constant of the GR receptor”. We changed “cortisol.. crosses Kd of the GR receptor” to “cortisol.. crosses the dissociation constant Kd of the GR receptor”.

P.4, l.-4 It is not clear to the readers what ‘our model’ is!!!?

Response: We changed “In our model” to “The HPA-CNS model gives the steady state solution”.

P.5, Figure 1. Remove the vector field or make it more visible and complete (it appears sparce on the phase plane).

Response: We have now revised these figures to include dense dynamical arrows.

P.6 Figure 1 caption. It is not clear how C) and D) actually are made, state if C) and D) correspond to high and low u.

Response: We now revised the caption of fig 1 “C and D used identical parameters except stress input u=0.75 and u=0.4 respectively. An analogous shift between 3 and 1 fixed points can occur when changing the CNS system parameters to represent genetic variants. “

P.8, Figure 2. Strange idea to change colors compared to figure 1 (this is also the case for figure 4). Please unify.

Response: We have now fixed this and used the same color scheme for all figures.

P.12, l.10 I suggest interchanging the word ‘minimal’ with ‘simple’.

Response: Agreed, changed “minimal” to “simple”.

P. 14, l.2 You refer to nicely to Karin et al. However, the equations (1-3) looked very much like some I saw year before this reference. Thus, I looked it up and to me it seems like Karin et al is inspired by Bangsgaard & Ottesen (2017) Patient specific modeling of the HPA axis related to clinical diagnosis of depression. Math. Biosciences, 287. The authors also have a reduction like the one your do leading to (4-6). I think it would be suitable to refer to this paper.

Response: Thank you for pointing out this oversight, indeed the Bangsgaard & Ottesen paper was the inspiration for this work and provides the fast timescale part of the model. We now cite it as suggested:

[43.	Bangsgaard, E. O. & Ottesen, J. T. Patient specific modeling of the HPA axis related to clinical diagnosis of depression. *Math. Biosci.* **287**, 24–35 (2017).](https://www.zotero.org/google-docs/?9q2wUs)

P.14, l.3 Put in bio-refs to support the claim or state that it is an assumption.

Response: We added references for these claims: “We denote the input signal to the HPA system as $u$[^67^](https://www.zotero.org/google-docs/?L6KN2S). This input is inhibited by the CNS activity $h$[^25–27^](https://www.zotero.org/google-docs/?lgx6NN), a novel feature of the present model.” Specifically we added:

[25.	Sahay, A. & Hen, R. Adult hippocampal neurogenesis in depression. *Nat. Neurosci.* **10**, 1110–1115 (2007).](https://www.zotero.org/google-docs/?9q2wUs)

[26.	Jacobson, L. & Sapolsky, R. The Role of the Hippocampus in Feedback Regulation of the Hypothalamic-Pituitary-Adrenocortical Axis*. *Endocr. Rev.* **12**, 118–134 (1991).](https://www.zotero.org/google-docs/?9q2wUs)

[27.	Weidenfeld, J., Ovadia, H., Weidenfeld, J. & Ovadia, H. *The Role of the Amygdala in Regulating the Hypothalamic-Pituitary-Adrenal Axis*. *The Amygdala - Where Emotions Shape Perception, Learning and Memories* (IntechOpen, 2017). doi:10.5772/67828.](https://www.zotero.org/google-docs/?9q2wUs)

[67.	Tsigos, C. & Chrousos, G. P. Hypothalamic–pituitary–adrenal axis, neuroendocrine factors and stress. *J. Psychosom. Res.* **53**, 865–871 (2002).](https://www.zotero.org/google-docs/?9q2wUs)

P.14, eq. (1) Can you provide the reader with a bio-reference saying that MR and GR act multiplicatively? If not emphasize that this is an assumption.

Response: We now write that this is an assumption. We now write in the revised Methods “We assume that the effects of MR and GR are multiplicative and use the same MR and GR expressions as done in Karin et al[^45^](https://www.zotero.org/google-docs/?2IrPl9).”

P.14, l.12-13. Are the expressions for MR(x_3) and GR(x_3) assumed or are these coming from data of some kinds?

Response: We now added a reference for the dose response curves of MR and GR, which were the basis of Karin et al functions: “MR inhibitory activity is non cooperative so that $MR(x_{3})=1+\frac{x_{3}}{K_{MR}}$*,* whereas GR inhibitory activity is Hill like with cooperativity coefficient 3[^45,68^](https://www.zotero.org/google-docs/?bH7rfS), so that $GR(x_{3})=1+(\frac{x_{3}}{K_{GR}})^{3}$*.* We note that at physiological cortisol levels, MR receptors tend to be nearly saturated whereas GR receptors are hardly activated [^39,69^](https://www.zotero.org/google-docs/?LHqUXS), namely $K_{MR}<<x_{3}<<$ $K_{GR}$ .” Specifically we added:

[39.	Andersen, M., Vinther, F. & Ottesen, J. T. Mathematical modeling of the hypothalamic–pituitary–adrenal gland (HPA) axis, including hippocampal mechanisms. *Math. Biosci.* **246**, 122–138 (2013).](https://www.zotero.org/google-docs/?9q2wUs)

[68.	Arterbery, A. *et al.* Evolution of ligand specificity in vertebrate corticosteroid receptors. *BMC Evol. Biol.* **11**, 14 (2011).](https://www.zotero.org/google-docs/?9q2wUs)

[69.	Koning, A.-S. C. A. M., Buurstede, J. C., van Weert, L. T. C. M. & Meijer, O. C. Glucocorticoid and Mineralocorticoid Receptors in the Brain: A Transcriptional Perspective. *J. Endocr. Soc.* **3**, 1917–1930 (2019).](https://www.zotero.org/google-docs/?9q2wUs)

P.14, l.16. I think you need to be specific about assuming GR=1 and MR=x_3 to arrive with the reduced system (4-6). Likewise, you should emphasize that these are only good approximations for K_MR<

Response: We agree, and have now added this clarification. On Pg 14 we write: “In this case we can approximate the MR and GR expressions - $MR(x_{3}) \approx x_{3}, GR(x_{3}) \approx1$*.* This provides a simpler set of equations for hormone levels (4-6):”

P. 14, l.21-22. Why the choice x_1 as growth factor for P and x2 for A? I would imagine that the hormone produced generates the growth, meaning that it should be x_ and x_3 respectively (this is common in many similar models). Give a bio-reference for this or emphasize these a (strange) assumptions!

Response: We now clarify that the choice of growth factors is based on experimental evidence. ACTH (x2) is the adrenal cortex trophic hormone and causes growth of the adrenal, and CRH is the growth factor for pituitary corticotrophs. We now clarify this in the text ”The growth factor of $P$ is $x_{1}$[^70,71^](https://www.zotero.org/google-docs/?iTUlsQ) and the growth factor of $A$ is $x_{2}$[^72,73^](https://www.zotero.org/google-docs/?Q7eIkT), as detailed in Karin et al[^45^](https://www.zotero.org/google-docs/?Rlv8LG)”, with the references:

70[.	Chronic Administration of Corticotropin-Releasing Factor Increases Pituitary Corticotroph Number* | Endocrinology | Oxford Academic.](https://www.zotero.org/google-docs/?9q2wUs) <https://academic.oup.com/endo/article/120/1/381/2540834>[.](https://www.zotero.org/google-docs/?9q2wUs)

[71.	The mitogenic effects of corticotrophin-releasing factor on the anterior pituitary gland of the rat in: Journal of Endocrinology Volume 118 Issue 2 (1988).](https://www.zotero.org/google-docs/?9q2wUs) <https://joe.bioscientifica.com/view/journals/joe/118/2/joe_118_2_012.xml>[.](https://www.zotero.org/google-docs/?9q2wUs)

[72.	Swann, H. G. THE PITUITARY-ADRENOCORTICAL RELATIONSHIP. *Physiol. Rev.* **20**, 493–521 (1940).](https://www.zotero.org/google-docs/?9q2wUs)

[73.	Lotfi, C. F. P. & de Mendonca, P. O. R. Comparative Effect of ACTH and Related Peptides on Proliferation and Growth of Rat Adrenal Gland. *Front. Endocrinol.* **7**, (2016).](https://www.zotero.org/google-docs/?9q2wUs)

P. 14, l.-3 ‘to a good approximation’ compared to what? Maybe just write ‘approximately’?

Response: We changed this to “approximately” as suggested.

P.14, l.-1-P.15, l.2. I think this is pseudo modeling, since you are really modeling the common MR and GR inhibition as a step function?

Response: We agree, and now clarify “We therefore, for simplicity, model the inhibitory effect of cortisol on the CNS as a step function, defined by $a+b\Theta(x_{3}>T)$*.* Using a Hill-type function instead of a step function leaves the conclusions unchanged but makes the analytical solution much more difficult.”

P.14-P.16 You may use the terms fast QSS and slow QSS consistently throughout this section!

Response: We believe that the slow QSS is actually a bona-fide steady state, since it is zero derivative for all 6 model equations including the fast and slow variables. We thus use steady-state and quasi steady state for the full steady state, and the fast variable QSS respectively throughout the paper.

P.15, eq (9) First term on the right-hand side is really alpha-beta*theta(x_3>T) for suitable choices of positive beta

Response: We agree. We used this form in the denominator to remind the reader that the step function is an approximation of a biochemical receptor (GR) dose response curve.

P.15, l.12 Is there an ‘of’ too much?

Response: we have now removed an extra ‘of’

P.15, l.17 ‘normalize’ do you mean \tilde{x}_1=x_1/x_st, etc. and then renaming \tilde{x}_1 as x_1 etc.?

Response: Yes. We now added a clarification: “We normalize our equations ${x̃}_{1}= \frac{x_{1}}{x_{1_{st}}}, {x̃}_{2}= \frac{x_{2}}{x_{2_{st}}}$, so the baseline level is now ${x̃}_{1_{st}}={x̃}_{2_{st}}=1$.”

P.15, l.17-23. Is sloppy hand-waving / non-rigid and intuitive. Either that should be crystal clear or done properly e.g., does a QSS approximation require a stability analysis!

Response: We have considered this again, and believe that the paragraph is accurate. We used the full solution of the six equations for the hormone x1 and x2 steady-state, to derive these conclusions on steady state gland sizes without a qss assumption.

P.16, l.3-6. This is not rigid but merely an illustration.

Response: We have now changed the phrasing to “demonstrate”

P.16, eq. (18) Should the power 1/2 be the power 2?

Response: It should be a power of ½, we double checked.

p.16, l. 12 ‘We’ after the period.

Response: Fixed.

P.16, l.13 Pst proportional to GR(x_3), but earlier you did assume GR(x_3)=1!

Response: We assumed GR(x3)=1 only to simplify the solution of the hormone quasi steady state and nullcline shape. For the compensation mechanism we use the more general and accurate equation (2-3) instead of (5-6). We added a clarification at the beginning of this section.

“”Our HPA-CNS model is the set of equations (eq 1,2,3,7,8,9) which describe the dynamics of hormones and glands in the HPA-CNS system.
In order to demonstrate the general features of the model and nullclines, we first use a simplified version (eq 4,5,6,7,8,9). When considering compensation mechanisms where the GR plays an important role, we use the full HPA-CNS model Eq (eq 1,2,3,7,8,9)”

*.*P.16. After reading the section I still do not know what your model exactly is. Please state the resulting model uniquely!

Response: We hope that the new clarification paragraph above addresses this concern.

P.16. Add a table with all parameter values used.

Response: we now added table 1:

“The nominal parameter values for the model are presented in table 1. These parameters provide 3 fixed points, and provide hormone levels equal to 1. The fold-change of each parameter required to bifurcate to a single fixed point are also shown. Some parameters cause a bifurcation both when raised and lowered, as represented by two fold-change values in the table.”

**Table 1 - HPA-CNS model nominal parameter values and sensitivities**

| Parameter | meaning | Nominal value | Fold-change for bifurcation to 1 fixed point |
| --- | --- | --- | --- |
| a | CNS inhibition of HPA at low cortisol | 1 | 0.5,1.5 |
| b | CNS inhibition of HPA at high cortisol | 1 | 0.5 |
| u | stress input | 1 | 0.75,1.5 |
| T | GR activation threshold in the CNS | 1.5 | 0.66, 1.33 |
| $a_{1}$ | CRH secretion parameter | 0.17 CRH*cortisol*h/min | 0.75, 1.5 |
| $b_{1}$ | CRH removal rate | 0.17/min[^39^](https://www.zotero.org/google-docs/?fZ0AwX) | 0.66, 1.33 |
| $a_{2}$ | ACTH secretion parameter | 0.035/min/(CRH*P) | 0.75, 1.5 |
| $b_{2}$ | ACTH removal rate | 0.035/min[^39^](https://www.zotero.org/google-docs/?DdrSCh) | 0.66, 1.33 |
| $a_{3}$ | cortisol secretion parameter | 0.0086/min/(ACTH*A) | 0.7, 1.45 |
| $b_{3}$ | cortisol removal rate | 0.0086/min[^39^](https://www.zotero.org/google-docs/?7yBqf3) | 0.68, 1.42 |
| $a_{P}$ | pituitary cell-mass production per unit CRH | 0.049/day/CRH | 0.85, 1.2 |
| $b_{P}$ | pituitary cell-mass removal rate | 0.049/day[^45^](https://www.zotero.org/google-docs/?V9eL9t) | 0.83, 1.17 |
| $a_{A}$ | adrenal cell-mass production per unit ACTH | 0.099/day/ACTH | 0.85, 1.2 |
| $b_{A}$ | adrenal cell-mass removal rate | 0.099/day[^45^](https://www.zotero.org/google-docs/?7zRFqH) | 0.83, 1.17 |
| $a_{h}$ | CNS production parameter | 0.047 h/day | 0.65, 1.35 |
| $b_{h}$ | CNS removal rate | 0.047/day | 0.74, 1.54 |

Reviewer #2: Review of Mizrachi et al, Major depressive disorder and bistability in an HPA-CNS toggle switch

This paper addresses the origin of slow (weeks long timescale) dynamics of major depressive disorder using a minimal mathematical model and dynamical systems approaches. The model focuses on the interaction between the hypothalamus-pituitary-adrenal axis (HPA) and the central nervous system (CNS), and specifically incorporates the important but overlooked phenomena by which activation can control hormone gland sizes. The authors focus on the role of mutual inhibition between CNS and HPA activity, the latter mediated by glucocorticoids.

Overall this work beautifully exemplifies ability of carefully crafted simplified dynamical models to provide powerful insights into the behavior of complex biological systems. It suggests an intriguing mechanism that explains how different setpoints in individuals can produce distinct (bistable vs. monostable) dynamics, how hysteretic effects can lead to extended timescales, and how specific disease-relevant perturbations could impact system dynamics. I anticipate it will have a major impact in the field of systems psychiatry, and will be of broader interest to researchers in systems biology, systems medicine, and psychiatry more generally. Given the increasing awareness and incidence of depression, especially among teens, I believe this work is also timely and potentially of interest beyond the academic and medical communities. The paper is beautifully and clearly written. I support acceptance in its current form.

We thank the reviewer for this endorsement.

However, below I suggest a few minor comments the authors may wish to consider.

Minor comments:

• Cosmetic: Some of the figures have oddly compressed fonts, or somewhat fuzzy (low resolution) graphics, possibly reflecting an issue with the image compression.

Response: fixed.

• It would be helpful to understand which parameters, if any, have the strongest impact on the qualitative predictions.

Response: We have now analyzed which parameters affect the existence of 1 or 3 fixed points. We use a nominal parameter set with 3 fixed points, and ask by what factor we need to change each parameter (individually) in order to bifurcate to 1 fixed point. We present the results in the new table 1.

• The authors may wish to consider making an animation of the nullcline analysis in which the reader could directly see how the nullclines and the system state “move” during various perturbations discussed in the text and, e.g. in Figure 2.

Response: We have now added an animation field to the paper using mathematica. The animations can be seen at this github address https://github.com/benron20/MDD-and-bistability-in-the-HPA-stress-axis.

**“Animation of phase portrait under parameter changes**

Animations, generated by Mathematica, of the phase portraits under changing u, a or b parameters can be found here:
<https://github.com/benron20/MDD-and-bistability-in-the-HPA-stress-axis>.”

Reviewer #3: This is a very important study that can help inform the treatment modalities for major depressive disorders, which afflict millions of people worldwide. By focusing on the HPA axis, which has a well documented role in MDD, the authors develop a mathematical model that describes the consequence of the HPA axis design (with two negative feedbacks and the resulting toggle switch) and importantly explain the requirement for long time scale of treatment to produce therapeutic effects. Although the study intentionally focused on one aspect of a complex problem, it provides valuable insights into the dynamics of HPA axis and explain previously poorly understood characteristics of MDD (requirement for prolonged stress) and its treatment (requirement for prolonged use of certain classes of anti-depressants).
I think overall this is a very valuable contribution with original insights that would benefit the community.

We thank the reviewer for this endorsement

**Have the authors made all data and (if applicable) computational code underlying the findings in their manuscript fully available?**
The [PLOS Data policy](https://journals.plos.org/ploscompbiol/s/materials-and-software-sharing) requires authors to make all data and code underlying the findings described in their manuscript fully available without restriction, with rare exception (please refer to the Data Availability Statement in the manuscript PDF file). The data and code should be provided as part of the manuscript or its supporting information, or deposited to a public repository. For example, in addition to summary statistics, the data points behind means, medians and variance measures should be available. If there are restrictions on publicly sharing data or code —e.g. participant privacy or use of data from a third party—those must be specified.

Reviewer #1: **No:** I could find any aailable code

Response: all code can be seen at this github address: https://github.com/benron20/MDD-and-bistability-in-the-HPA-stress-axis.

Reviewer #2: Yes

Reviewer #3: Yes
